# Ameliorative Effects of Blue LED Irradiated and Non-Irradiated Fenugreek Seed Extracts on Murine Trichinosis

**DOI:** 10.1007/s11686-025-01045-8

**Published:** 2025-06-02

**Authors:** Zeinab R. Hassan, Eman M. Mahmoud, Yasmeen M. Shaaban, Shiamaa Zakaria Elshora, Doaa E. A. Salama, Ranya M. Abdelgalil, Mona T. Koullah, Nora Seliem, Marwa H. Sedira, Shimaa A. Mohamed, Mai Ali Mohammad Etewa, Sara Nagdy Mahmoud Mousa, Ahmed Shaban Abdelmonsef Abdelmaksoud, Shimaa Attia Atta, Marwa Zakaria, Shimaa R. Emam, M. Hassan, Galal Khamis

**Affiliations:** 1 Departments of Parasitology, Faculty of Medicine for Girls, Al-Azhar University, Cairo, Egypt; 2 Department of Histology, Faculty of Medicine for Girls, Al-Azhar University, Cairo, Egypt; 3 Department of Pathology, Faculty of Medicine for Girls, Al-Azhar University, Cairo, Egypt; 4 Department of Anatomy and Embryology, Faculty of Medicine for Girls, Al-Azhar University, Cairo, Egypt; 5 Department of Medical Biochemistry and Molecular Biology, Faculty of Medicine for Girls, Al-Azhar University, Cairo, Egypt; 6 Department of Physiology, Faculty of Medicine for Girls, Al-Azhar University, Cairo, Egypt; 7 Department of Immunology, Theodor Bilharz Research Institute, Cairo, Egypt; 8 Department of Educational Central Lab, Women College, Ain Shams University, Cairo, Egypt; 9 Department of Medical Biochemistry and Molecular biology, School of Medicine, Badr University in Cairo (BUC), Cairo, Egypt; 10 Department of Pharmaceutical Production Technology, Faculty of Applied Health Science Technology, October Technological Univerisity, Giza, Egypt; 11 Department of Pharmacology, Faculty of Veterinary Medicine, Cairo University, Giza, Egypt; 12 Department of Laser Application in Metrology, Photochemistry and Agriculture (LAMPA), National Institute of Laser Enhanced Sciences (NILES), Cairo University, Giza, Egypt; 13 Departments of Parasitology, Benha National University (BNU) Qalyubia, Obour, Egypt; 14 Department of Anatomy and Physiology, College of Medicine, Imam Mohammad Ibn Saud Islamic University (IMSIU), Riyadh, Saudi Arabia; 15 Department of Medical Physiology, Damietta Faculty of Medicine, Al-Azhar University, Damietta, Egypt; 16 Department of Pathology, School of Medicine, Badr University in Cairo (BUC), Cairo, Egypt

**Keywords:** Albendazole, Apoptotic gene, *BAX*, Fenugreek, IFN-γ, Oxidative stress markers, TGF-β, Trichinosis

## Abstract

**Background:**

Trichinosis is a severe parasitic disease with a wide distribution and potential to affect humans. Available chemotherapeutic agents exhibit limited efficacy and are associated with numerous side effects. This study evaluated the ameliorative effects of blue LED irradiated and non-irradiated fenugreek seed extracts on experimental trichinosis.

**Methods:**

Eighty-four mice were divided into seven groups; each further subdivided into intestinal and muscular phases (six mice per subgroup): non-infected non-treated controls, infected non-treated controls, infected albendazole-treated, infected non-irradiated fenugreek extract-treated, infected blue LED irradiated fenugreek extract-treated, infected non-irradiated fenugreek and albendazole-treated, and infected blue LED irradiated fenugreek and albendazole-treated. Mice were sacrificed on the 7th-day post-infection for the intestinal phase and the 40th day for the muscular phase. Small intestine, muscle tissues, and serum samples were collected to assess parasitic load, histopathological changes, TGF-β immunohistochemical expression, serum IFN-γ levels, oxidative stress markers (MDA, nitrate/SOD, and catalase), and *BAX* gene expression as an apoptotic marker. Metabolomic profiling of extract was pursued to spot differential expression of metabolites.

**Results:**

Study outcomes demonstrated that blue LED irradiated and non-irradiated fenugreek seed extracts combined with albendazole exhibited superior efficacy in reducing adult and larval burdens, improving pathological changes, decreasing IFN-γ levels, mitigating oxidative stress (reduced MDA and nitrate along with elevated SOD and catalase), and downregulating *BAX* expression. The observed metabolic differences were primarily driven by the upregulation of steroids, downregulation of most alkaloids, and dysregulation various flavonoids in the irradiated extract.

**Conclusion:**

Blue LED irradiated and non-irradiated fenugreek seed extracts can enhance albendazole's activity against trichinosis.

## Introduction

Trichinosis is a widely distributed zoonotic parasitic disease, and the risk of human infection arises from consuming viable *Trichinella* larvae embedded in raw or undercooked pork meat [1]. Adult and larval stages of *Trichinella* spp. develop within the same host, resulting in intestinal and muscular phases. Larvae released from ingested encapsulated cysts or free non-encapsulated larvae depending on the species invade the intestinal mucosa and mature into adult stages. Following mating, newborn larvae migrate to striated muscles, becoming encysted, forming nurse cells that provide protection and facilitate survival [2].

One of the current chemotherapeutic drugs used to treat trichinosis is benzimidazole derivatives as albendazole (ABZ), though its efficacy is mainly limited to adult worms and non-encysted larvae [3]. Additionally, ABZ has been associated with several side effects, including gastrointestinal disturbances, hepatotoxicity, and teratogenicity [4]. Resistance to ABZ has been reported in some *Trichinella* strains, particularly during the muscular phase [3, 5].

Fenugreek (*Trigonella foenum-graecum*) is a plant utilized as both fodder and spice, with numerous medicinal applications. It has demonstrated gastro-protective, hypocholesterolemic, hypoglycemic, anticancer, anti-malarial, anti-allergic, antibacterial, and antiviral properties [6]. A fenugreek seed extract has antioxidant effects, regenerating damaged heart and skeletal muscles [7]. Phytochemical analysis of fenugreek seeds has revealed the presence of alkaloids, saponins, and flavonoids [8].

Blue light-emitting diode (LED) irradiation is an eco-friendly method that influences plant growth and enhances the bioactive compounds of plants, thereby increasing their medicinal properties [9, 10].

Therefore, the current study aimed to evaluate the effects of blue LED irradiated fenugreek (FNK R) and non-irradiated fenugreek (FNK) extracts adult *Trichinella spiralis* in the intestines and of larvae in muscles, including the inflammatory and degenerative changes in experimentally infected mice.

## Materials and Methods

### Study Design

**Fig. 1:**
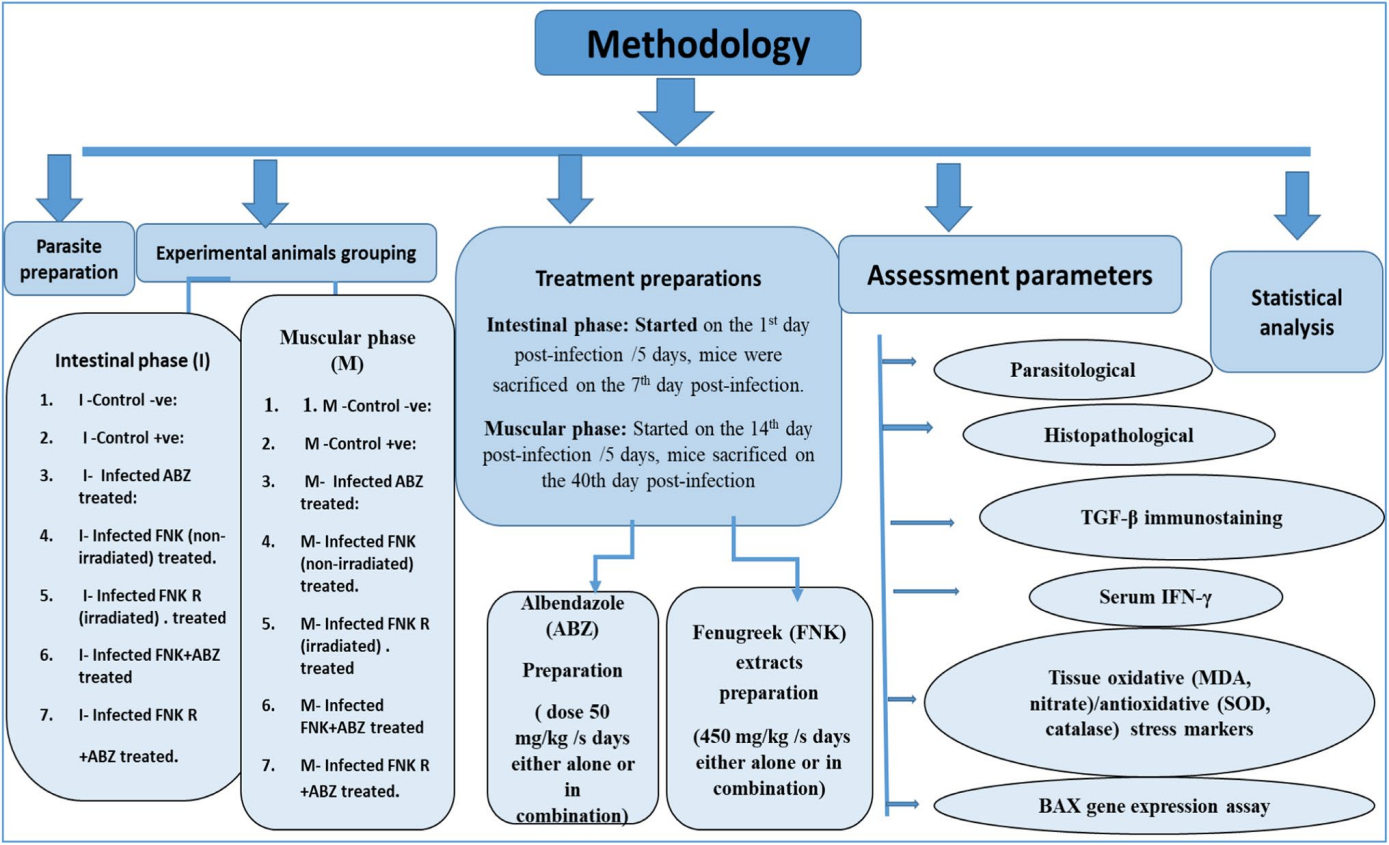
Methodology

The current study was conducted on eighty-four Swiss albino male laboratory-bred mice weighing 20–25 g and aged 4–6 weeks. The animals were housed and maintained in the Theodor Bilharz Research Institute's (TBRI) animal house per ethical guidelines for animal handling. Treatment commenced on the 1 st day post-infection for the intestinal phase and continued for 5 days, after which mice were sacrificed on the 7 th day post-infection under intraperitoneal anesthesia (100 units/ml heparin and 500 mg/kg thiopental). For the muscular phase, treatment began on the 14 th day post-infection and continued for 5 days, with mice sacrificed on the 40 th day post-infection. Tissues and sera from both phases were collected for parasitological, histopathological, immunohistochemical, immunological, oxidative tissue marker, and gene expression assessments (Fig. 1).

### Parasite and Experimental Animals

*T. spiralis* larvae were kindly provided by TBRI animal house that maintain the cycle through sequential passages of the parasite strain in mice. Dissected muscles of infected mice were incubated at pepsin-hydrochloride 1%, then the mixture was washed and filterated several times. The 1 st stage larvae were collected from the sediment and mice infections were performed through orogastric gavage using insulin syringe with approximately 200 motile larvae per mouse [11].

Mice were divided into seven groups, each further subdivided into I (intestinal phase) and M (muscular phase) subgroups (six mice each), as follows: I-control -ve and M-control -ve as non-infected non-treated controls, I-control + ve and M-control + ve as infected non-treated controls, I-ABZ and M-ABZ as infected albendazole-treated mice, I-FNK and M-FNK as infected FNK extract-treated mice, I-FNK R and M-FNK R as infected irradiated FNK extract-treated mice, I-FNK + ABZ and M-FNK + ABZ as infected FNK and albendazole-treated mice, and I-FNK R + ABZ and M-FNK R + ABZ as infected FNK R and albendazole-treated mice.

### Treatment Preparation

ABZ (albendazole tablets, Pharma Cure Pharmaceuticals, Cairo, Egypt) in powder form and dissolved in distilled water, and a dose of 50 mg/kg was administered orally to the selected animal groups in both the intestinal and muscular phases [12, 13].

### Fenugreek Seeds Collection and Irradiation

Fenugreek seeds were procured from a local market in Egypt, purified manually of impurities, and divided into two groups of 400 g each. The FNK group remained non-irradiated, while the FNK R group was irradiated using Seeds Irradiation Instrument (SII), Model MHP LED Blue & Red, BRLED174-25-022. MADE IN EGYPT. The device adopts irradiation using Light Emitting Diodes (LEDs), blue LED biostimulation was investigated in current research at 455 nm peak 0.3–0.4 mW/cm^2^, dosage received by seeds was 1.5 mJ/cm^2^. The device consists of two transparent drawers (seed drawer 1 and seed drawer 2–90 × 45 cm). Blue treated seeds were placed onto irradiation shelves in a flat arrangement to ensure that all seeds received the same amount of irradiation dosage, irradiance measured using UT383, UNI-T luxmeter. After irradiation, both groups were planted in pots containing a clean planting medium and were irrigated regularly for two weeks. The seeds were subsequently collected, cleaned, and air-dried to prepare plant extracts from FNK and FNK R seedlings.

Fresh FNK and FNK R seeds (400 g each) were washed with water, dried at 50 ± 2 °C by spreading them in a thin layer on a polythene sheet, and then moderately pulverized. The dry powdered plants (150 g and 165 g, respectively) were extracted via percolation in 80% methanol for 24 h, repeated several times until complete exhaustion. The solvent was evaporated under reduced pressure using a rotary evaporator (Büchi, Switzerland) at a low temperature not exceeding 50ºC. The yield was 23 g for FNK and 20 g for FNK R.

The extracts were stored at −4ºC until use. Before the experiments, the extracts were freshly suspended in distilled water with a few drops of Tween-80. Selected mice from intestinal and muscular phases received 450 mg/kg/day from each extracts orally for 5 days [7].

### Assessment Parameters

#### Parasitological Assessment

The small intestines were removed and processed for mice in the intestinal phase following the method described by Hassan *et al.* (2024) [14] and examined to count the adult worm burden. Muscular tissues from mice in the muscular phase were dissected, sliced, digested, filtered, then washed and centrifugated, and the sediment was examined to calculate the larval burden [15].

### Histopathological Assessment

Specimens of the small intestine (~ 1 cm at the junction of the proximal 1/3 and distal 2/3) and muscle (diaphragm, tongue, and thigh) from different phases were collected, fixed (using 10% formalin), dehydrated (using ascending grades of alcohol), cleared (using xylol), followed by paraffin embedding, and sections of 4 µm thickness were stained with hematoxylin and eosin (H&E) stain [16].

### Immunohistochemical Assessment

Immunostaining of muscular paraffin sections was performed using a transforming growth factor-beta (TGF-β) monoclonal antibody (DAKO, USA). Immunohistochemical analysis of the examined tissue was conducted using light microscopy. Immunopositive TGF-β stained cells appeared brown, and their staining intensity was calculated and scored as follows: negative (0) for zero stained cells or less than 3%, light (1 +) for 3–33% stained, medium (2 +) for 34–66% stained, and severe (3 +) for more than 66% stained [17].

### Serum Immunological Assessment

Serum was separated from blood samples collected during both the intestinal and muscular phases and stored at −20 °C until the IFN-γ levels were evaluated using IFN-γ ELISA kits (SUNLONG, China). The optical density was measured at 450 nm, and the sample concentration was calculated using the standard curve [18].

### Tissue Oxidative/Antioxidative Stress Markers Assessment

Malondialdehyde (MDA) and nitrate, as oxidative markers, along with superoxide dismutase (SOD) and catalase, as antioxidant enzymes, were measured in muscular tissue homogenates using available kits (Bio Diagnostic, Egypt) [19].

### BAX Gene Expression Assay

According to the manufacturer's instructions, total RNA was extracted from muscular tissue lysates using a Direct-zol RNA Miniprep Plus kit (ZYMO RESEARCH CORP., USA). Reverse transcription of the extracted RNA (cDNA synthesis) was performed using the Superscript IV One-Step RT-PCR Kit (Thermo Fisher Scientific, Waltham, MA, USA), followed by PCR amplification in a single reaction tube using *BAX* gene primers (5′-ACTCCCATTCTTCCACCTTTG-3′ and 5′-CCCTGTTGCTGTAGCCATATT-3′) and the internal control primers for *GAPDH* (5′-TGGATTTGGACGCATTGGTC-3′ and 5′-TTTGCACTGGTACGTGTTGAT-3′).

The prepared reaction mix samples were analyzed using real-time PCR (StepOne, Applied Biosystems, Foster City, USA). The data were expressed in cycle threshold (Ct). Relative quantification of the *BAX* gene was calculated and normalized to the housekeeping gene (*GAPDH*) using the delta-delta Ct (∆∆Ct) method, and relative gene expression was determined using the 2^−∆∆Ct^ formula [20].

### Statistical Analysis

SPSS version 24 was used to analyze the collected data, expressed as mean ± standard deviation (SD). The Student's t-test and one-way ANOVA were used to compare significant differences between two groups and more than two groups. A *p*-value < 0.05 was considered the cutoff for significance.

## Results

### Parasitological Count of Intestinal Adult and Muscular Larva Parasites

The counts of adult and larval stages showed a significant reduction after treatment in both phases. It was observed that the administration of ABZ alone or combined with different FNK extracts resulted in a 100% reduction in the intestinal phase (Table 1).

**Table 1:** Count of adult intestinal worm and muscular larvae of *T. spiralis* among different mice groups

Intestinal Adult worms count				Muscular Larvae count			
Groups	Mean ± SD	*T-test/* *P* value	Percent of Reduction	Groups	Mean ± SD	*T-test* */P* value	Percent of Reduction
I-Control + ve	83.75 ± 5.85	–	–	M-Control + ve	99.25 ± 8.42	–	–
I-ABZ	0	35.07/ *p* < 0.001^*^	100%	M-ABZ	35.25 ± 6.5	14.74/*p* < 0.001^*^	64.5%
I-FNK	42.25 ± 5.91	12.22/ *p* < 0.001^*^	49.6%	M-FNK	49.25 ± 2.99	13.71/*p* < 0.001^*^	50.4%
I-FNK R	32.5 ± 3.42	18.53/ *p* < 0.001^*^	61.2%	M-FNK R	34.25 ± 3.3	17.61/*p* < 0.001^*^	65.5%
I-FNK + I-ABZ	0	35.07/ *p* < 0.001^*^	100%	M-FNK + M-ABZ	18 ± 1.83	23.1/*p* < 0.001^*^	81.9%
I-FNK R + I-ABZ	0	35.07/ *p* < 0.001^*^	100%	M-FNK R + M-ABZ	10.5 ± 1.29	25.52/*p* < 0.001^*^	89. 4%
ANOVA F- test *P* value	504.81 0.000*			ANOVA F- test *P* value	260.83 0.000*		

I&M Control + ve, Infected non-treated control; I& M_-_ ABZ, Infected ABZ treated mice; I& M_-_ FNK, Infected non-irradiated FNK extract treated; I& M_-_ FNK R, Infected irradiated FNK extract; I& M_-_ FNK + ABZ, Infected FNK and ABZ treated t; I& M_-_ FNK R + ABZ, Infected FNK R and ABZ treated

^*^P < 0.05; Significant

### Histopathological Results

**Fig. 2:**
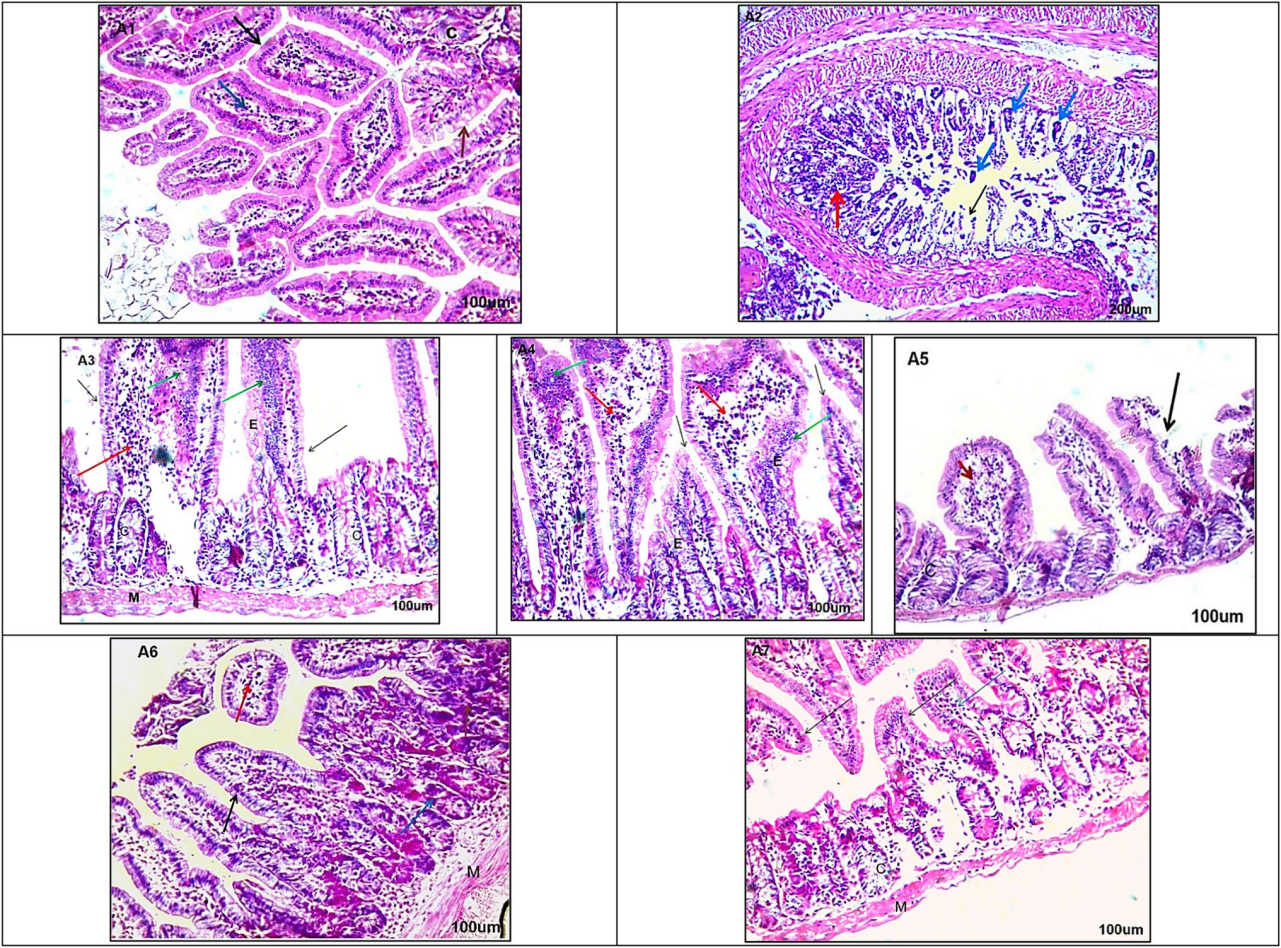
Sections of small intestine stained with H&E from studied groups (A1–A7) **A1** Non-infected non-treated control (I-Control -ve) showing normal architecture of intestinal villi which appear regular, and lined by simple columnar epithelium (black arrow) with goblet cells (brown arrow) with a core of connective tissue (blue arrow) normal architecture of crypt (C shape)100 ×. **A2** Infected non-treated control (I-Control + ve) showing cut sections in *T. spirals* adults appear in the core of villi (blue arrows), atrophic changes, sloughing and distortion villi (black arrow), dense inflammatory cellular infiltrate (red arrows) (100 ×). **A3** Infected ABZ treated (I- ABZ) (200 ×) showing edema (E shape), broadening of vilii, some of them lined by normal columnar cells (black arrows), the others showingdegenerative changes on large area lined by stratified epithelium; (hyperplsia) (green arrows) dense inflammatory cellular infiltrate (red arrows). normal appearance of crypts (C shape) and normal appearance of musculosa (M shape) (200 ×). **A4** Infected FNK extract treated (I- FNK) showing showing edema (E shape), broadening of vilii, some of them lined by normal columnar cells (black arrows), the others showingdegenerative changes on certain area lined by stratified epithelium; (hyperplsia) (green arrows) dense inflammatory cellular infiltrate (red arrows) (200 ×). **A5** Infected radiated FNK extract (I- FNK R) showing sloughing and shortening of the villi (black arrows), moderate inflammatory cellular infiltrate (red arrows) (200 ×). **A6** Infected FNK and ABZ treated (I-FNK + ABZ), showing normal appearance of cut sections of villi (black arrow), relative nuclear changes on epithelial lining crypts; pyknosis of their nuclei (blue arrow), minimal inflammatory cellular infiltrate (red arrows) with normal appearance of musculosa (M shape) (200 ×). **A7** Infected FNK R and ABZ treated (IN- FNK R + ABZ) showing marked improvement; villi (black arrows), connective tissue core (green arrow), and crypts (C shape) musculosa (M shape) all appears near normal with no histopathological changes (200 ×)

Sections of the small intestine at the intestinal phase revealed *T. spiralis* adults with dense inflammatory cellular infiltrate and marked atrophic changes in the villi in the infected non-treated group (I-control + ve) (Fig. 2 A2). In contrast, other intestinal sections from various treated mouse groups showed variable degrees of healing and restoration of histopathological changes (Fig. 2 A3-A7), with the most marked improvement observed in the mice group treated with combined FNK R and ABZ (I-

FNK R + ABZ) (Fig. 2 A7), followed by the combined FNK and ABZ treatment (I-FNK + ABZ) (Fig. 2 A6).

**Fig. 3:**
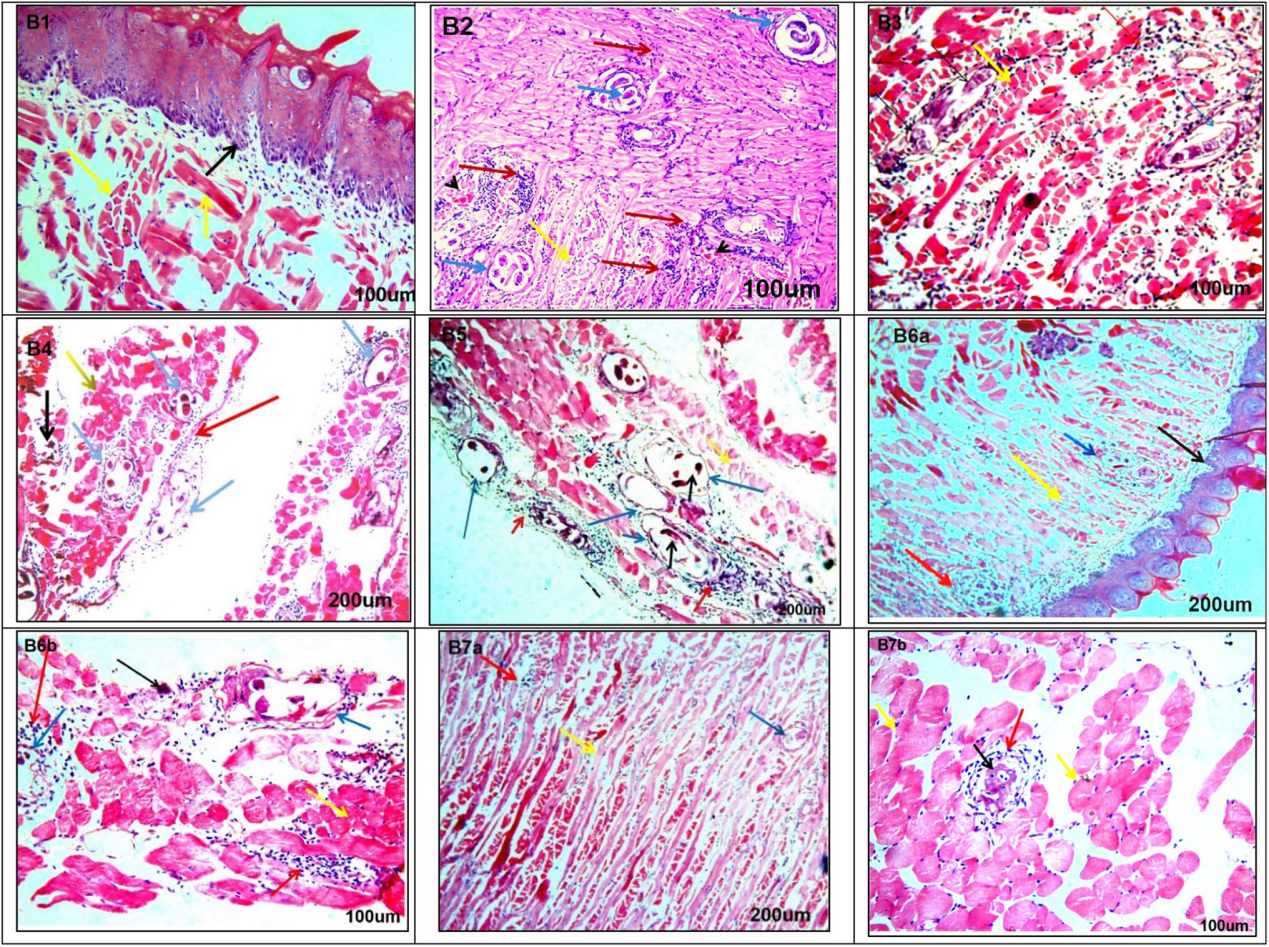
Sections of tongue muscle among all mice groups stained with H&E; **B1** Non-infected non-treated control (M-Control -ve) showing normal architecture of tongue papillae with connective tissue core (black arrow), normal architecture of muscle fibers which run in a different direction have acidophilic cytoplasm and peripherally situated nuclei (yellow arrows) (200 ×). **B2** Infected non-treated control (M-Control + ve) revealed the presence of a massive number of encysted *T. spiralis* larvae surrounded by an intact well-formed capsule (blue arrows), some degenerative changes on muscle fibers (yellow arrow) and a massive number of inflammatory cells infiltrating muscle bundles and surrounding the encysted larvae (red arrows) also there are many areas of hemorrhage (arrowheads) in between muscle fibers 200x. **B3** Infected ABZ treated (M-ABZ) showing normal appearance of muscle fibers (yellow arrow) but there are several encysted *T. spiralis* larvae with some degenerative changes of larvae (blue arrows) and a moderate number of inflammatory cells infiltrating muscle bundles (red arrow) 100x. **B4**Infected non-irradiated FNK extract treated (M- FNK) showing some degenerative changes on the muscle fibers with loss of their nuclei and widely separated from each other (yellow arrow) but there are a massive number of encysted *T. spirals* larvae with some degenerative changes (black arrow) thin non-intact capsule (blue arrows) and moderate number of inflammatory cells infiltrating muscle bundles and surrounding wall of larvae (red arrow) 200x. **B5** Infected irradiated FNK extract (M- FNK R) showingmarked degenerative changes of muscle fiber (yellow arrow), encysted *T. spirals* larvae with marked degenerative changes of larvae (black arrows), thin non-intact capsule (blue arrows) intense inflammatory cellular infiltration (red arrows) encircle the capsule and penetrate it, even adhering to the larva (red arrow) 200X. **B6** Infected FNK and ABZ treated (M- FNK + ABZ), B6a; showing normal architecture of tongue papillae (black arrow) normal appearance of muscle fibers (yellow arrow) but there are little number of encysted *T. spiral's* larvae with marked degenerative changes(blue arrow) with moderate number of inflammatory cells in between muscle fibers (red arrow) × 100; **B6b** showing minimal degenerative changes of muscle fibers (yellow arrow), marked degenerative changes on larvae (black arrow) and incomplete destruction of the capsule encircled (blue arrow), with moderate number of inflammatory cells that alsoinfiltrating muscle bundles (red arrow) 200X. **B7** Infected FNK R and ABZ treated (M- FNK R + ABZ); **B7a** showing normal appearance of muscle fibers (yellow arrow) with the presence of a little number of encysted *T. spiralis* larvae (blue arrow),and mild inflammatory cells (red arrow) 100x. **B7b** showing normal appearance of muscle fibers (yellow arrow) marked degenerated larvae with loss of their capsule (black arrow) with mild inflammatory cells (red arrow) 200x

On the other hand, muscle sections recovered from mice at the muscular phase revealed several encysted *T. spiralis* larvae surrounded by well-formed capsules and encircled by a massive number of inflammatory cell infiltrates, with some degenerative changes in muscle fibers. Areas of hemorrhage and dense inflammatory cell infiltration in muscle bundles were observed in the infected non-treated group (M-control + ve) (Fig. 3B2). Various treated mouse groups exhibited different degrees of degeneration of the encysted *T. spiralis* larvae and the surrounding capsule (Fig. 3B3-B7). However, the most notable improvement in muscle fibers and marked degeneration of the larvae was observed after treatment with combined FNK R and ABZ (M-FNK R + ABZ) (Fig. 3B7a & b), followed by the combined FNK and ABZ treatment (M-FNK + ABZ) (Fig. 3B6a & b).

### Immunohistochemical Results

**Fig. 4:**
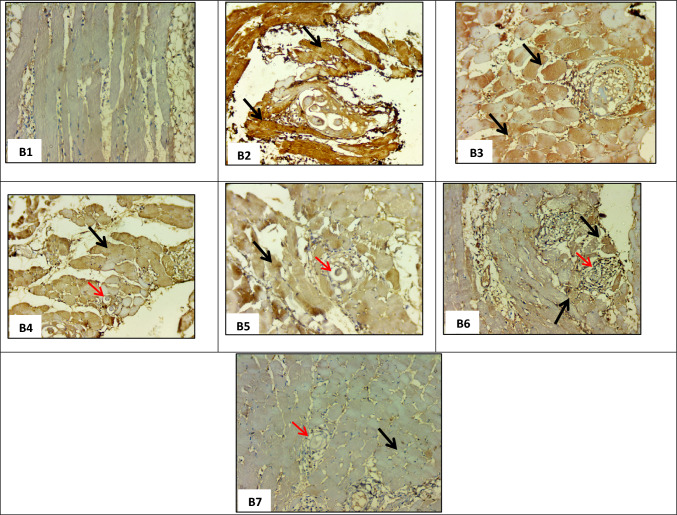
Immunohistochemical staining of TGF-β expression in muscle sections among mice groups at muscular phase (× 200); **B1** Non-infected non treated control (M-Control -ve) showing negative expression (0); **B2** Infected non treated control (M-Control + ve) showing severe expression of TGF-β in 70% of cells (+ 3) (black arrows); **B3** Infected ABZ treated (M_-_ ABZ) showing medium expression of TGF-β in 55% of cells (+ 2) (black arrows); **B4** Infected non-irradiated FNK extract treated (M_-_ FNK) showing medium expression of TGF-β in 55% of cells (+ 2) (black arrow), and degenerated cysts (red arrow); **B5** Infected irradiated FNK extract (M- FNK R) showing medium expression of TGF-β in 55% of cells (+ 2) (black arrow); and degenerated cysts (red arrow); **B6** Infected FNK and ABZ treated (M- FNK + ABZ) showing light expression of TGF-β in 30% of cells (+ 1) (black arrow); and degenerated cysts (red arrow); **B7** Infected FNK R and ABZ treated (M_-_ FNK R + ABZ) showing light expression of TGF-β in 17% of cells (+ 1) (black arrow) and degenerated cysts (red arrow)

Results revealed severe muscular expression of TGF-β in the infected non-treated group (M-control + ve) (Fig. 4B1). Single administration of ABZ (M-ABZ), FNK (M-FNK), and FNK R (M-FNK R) resulted in medium expression of TGF-β (Fig. 4B3–B5). On the other hand, mice groups that received ABZ with FNK (M-FNK + ABZ) and ABZ with FNK R (M-FNK R + ABZ) showed light expression of TGF-β, which was observed among various infected treated mouse groups (Fig. 4B6 and B7).

### Results of Serum IFN-γ Assessment

**Fig. 5:**
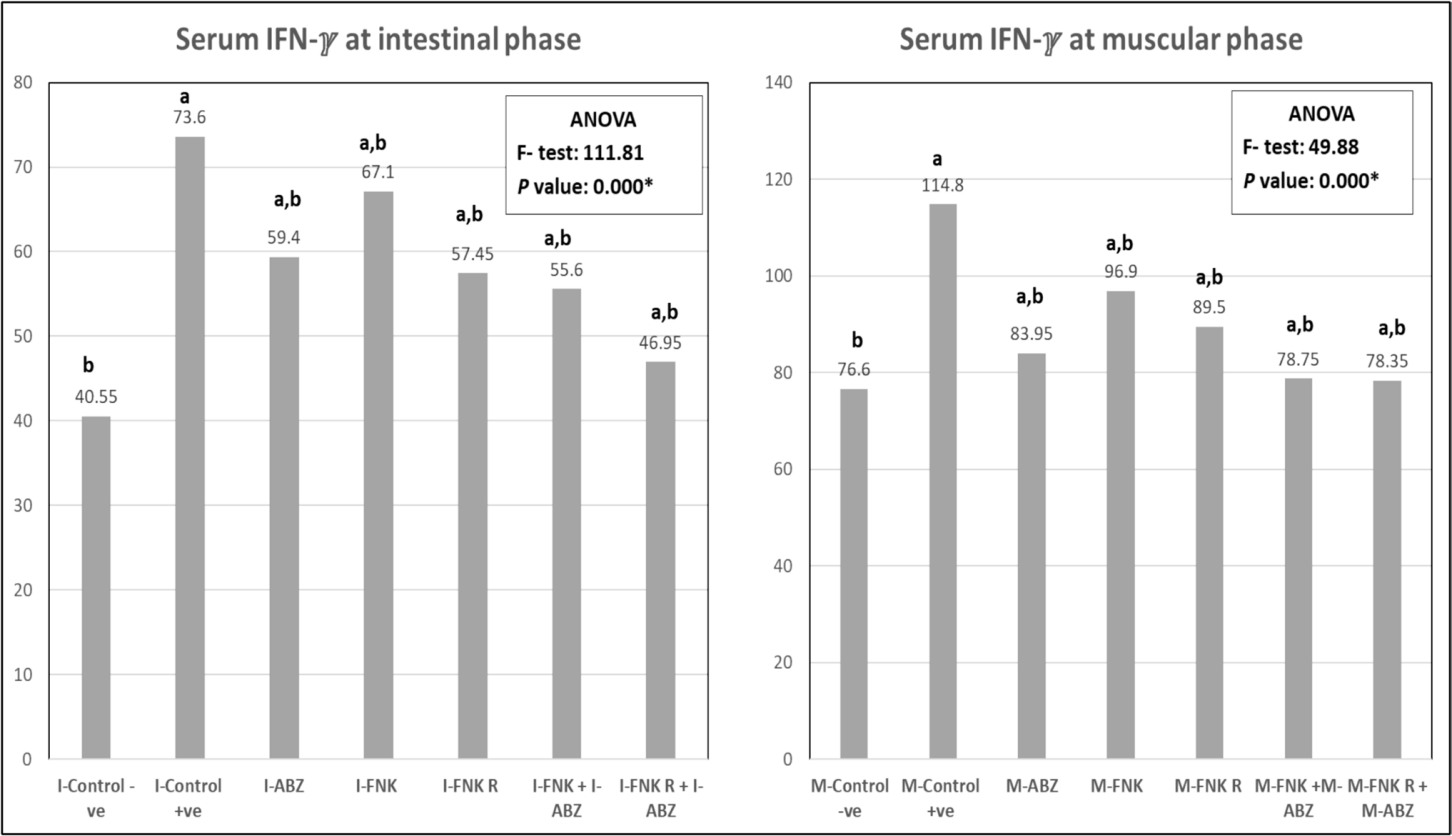
Serum level of IFN-ℽ among different mice groups. [I&M Control -ve: Non infected non treated control I&M Control + ve: Infected non-treated control, I& M_-_ ABZ: Infected ABZ treated mice, I& M_-_ FNK; Infected non-irradiated FNK extract treated, I& M_-_ FNK R; Infected irradiated FNK extract, I& M_-_ FNK + ABZ; Infected FNK and ABZ treated t, I& M_-_ FNK R + ABZ; Infected FNK R and ABZ treated. Significant when *P* < 0.05*; **a** refers to compared to control –ve; **b** refers to compared to control + ve)]

Results revealed a significant decrease (*p* < 0.05) in serum levels of IFN-γ in the different treated groups compared to the infected non-treated group (control + ve) in both the intestinal and muscular phases(Fig. 5).

### Results of Tissue Oxidative Stress Markers Assessment

**Fig. 6:**
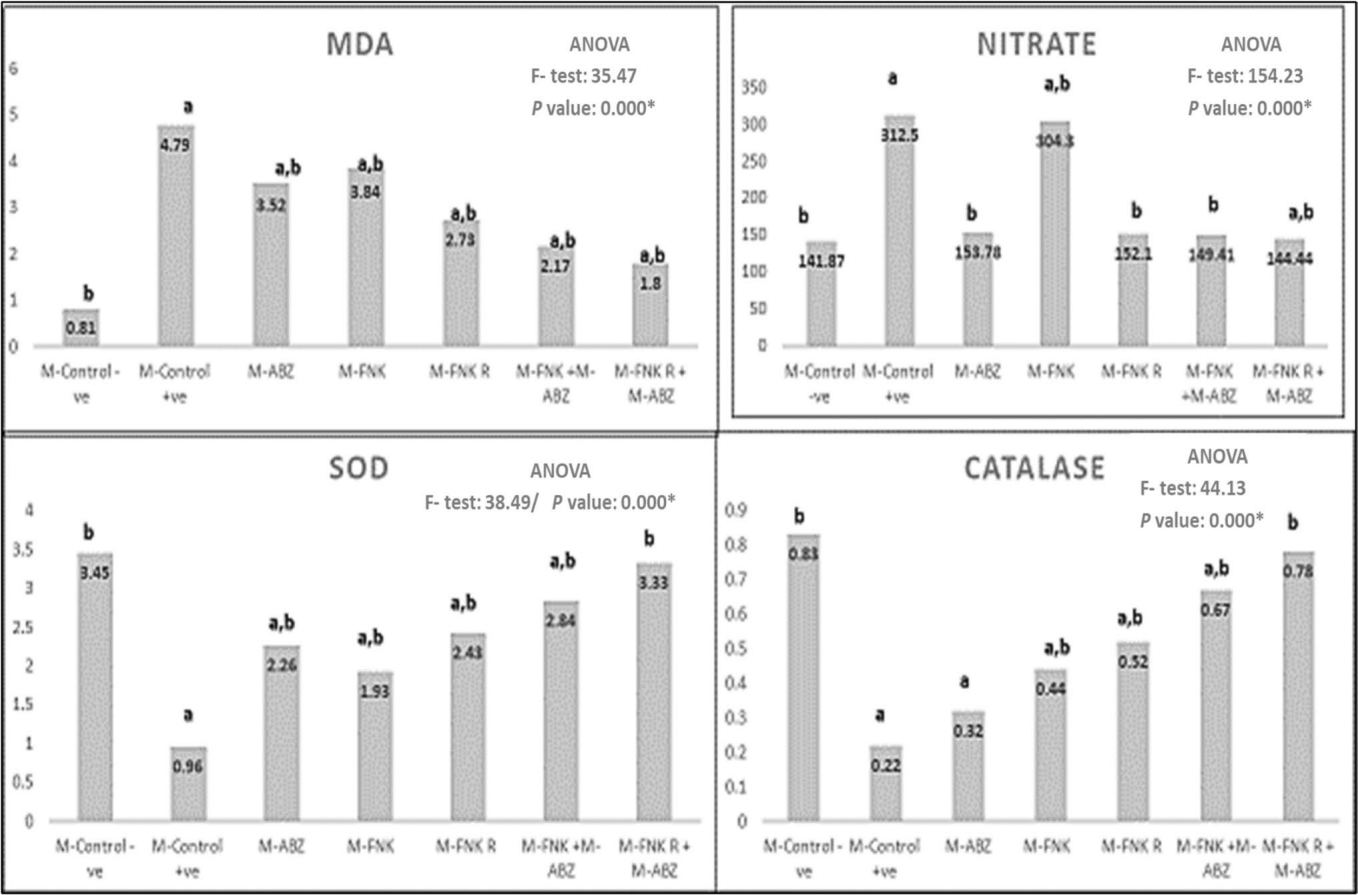
Levels of different tissue oxidative stress markers. [M -Control -ve: Non infected non treated control, M- Control + ve: Infected non-treatedcontrol, M_-_ ABZ: Infected ABZ treated mice, M_-_ FNK; Infected non-irradiated FNK extract treated, M_-_ FNK R; Infected irradiated FNK extract, M_-_ FNK + ABZ; Infected FNK and ABZ treated t, M_-_ FNK R + ABZ; Infected FNK R and ABZ treated. Significant when *P* < 0.05*; **a** refers to compared to control –ve; **b** refers to compared to control + ve)]

Muscular tissue levels of oxidative enzymes, including MDA and nitrate, showed a significant increase after infection in all mouse groups, both treated and non-treated, compared to the non-infected non-treated group (control -ve). However, a significant decrease (*p* < 0.05) was observed in all treated groups compared to the infected non-treated group (control + ve). Conversely, a significant decrease (*p* < 0.05) in antioxidant enzymes, including SOD and catalase, was noted in all infected mice, both treated and non-treated, compared to the non-infected non-treated group (control -ve), except in mice treated with combined ABZ and FNK R. Additionally, all treated mice showed a significant increase in antioxidant enzymes compared to the infected non-treated group (control + ve), except those treated with ABZ alone (Fig. 6).

### BAX Gene Expression Assay Results

**Fig. 7:**
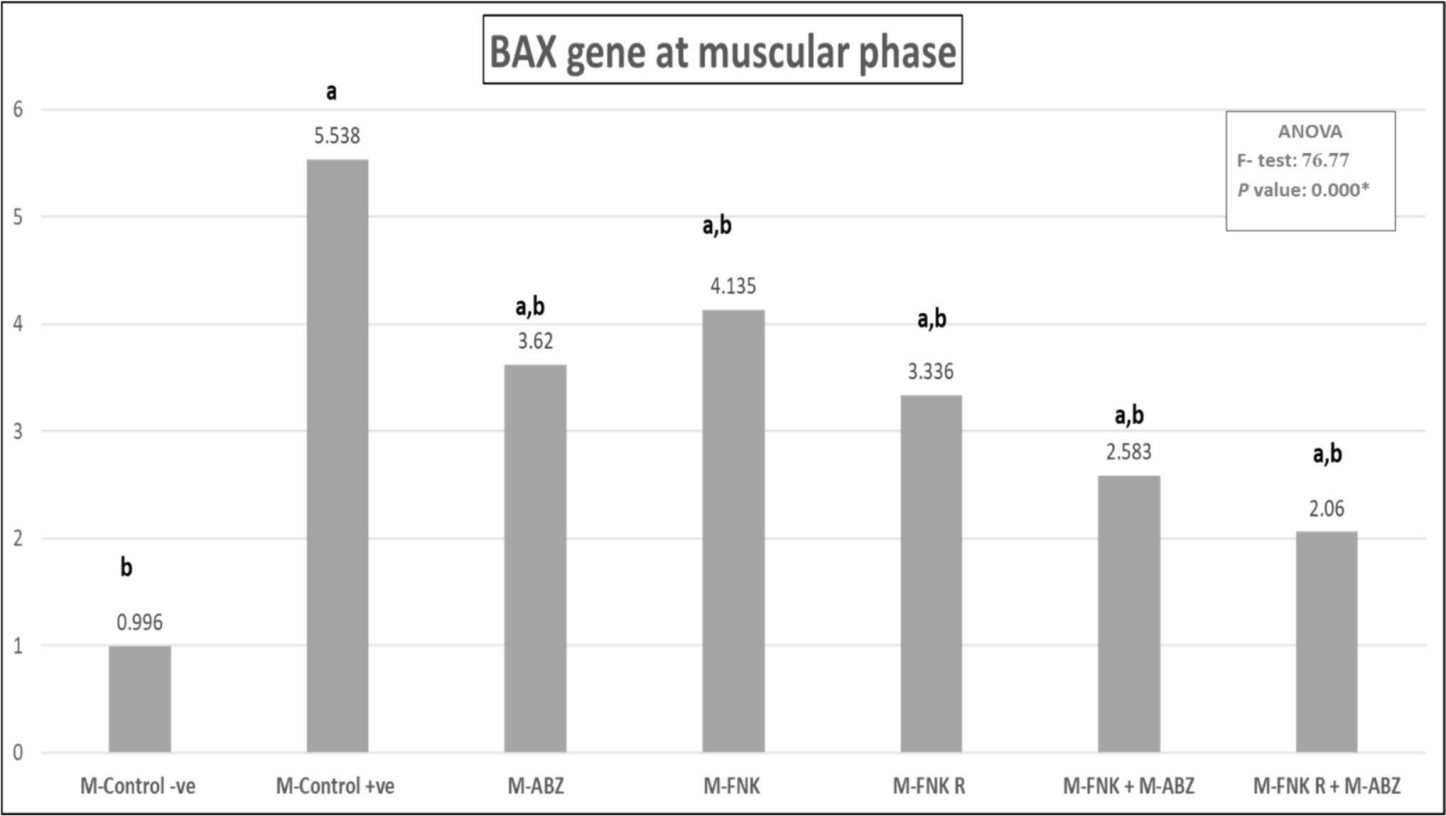
Different levels of BAX gene expression. [M-Control -ve: Non infected non treated control, M-Control + ve: Infected non treated control, M_-_ ABZ: Infected ABZ treated mice, M_-_ FNK; Infected non-irradiated FNK extract treated, M_-_ FNK R; Infected irradiated FNK extract, M_-_ FNK + ABZ; Infected FNK and ABZ treated t, M_-_ FNK R + ABZ; Infected FNK R and ABZ treated. Significant when *P* < 0.05*; **a** refers to compared to control –ve; **b** refers to compared to control + ve)]

There was a significant downregulation (*p* < 0.05) in *BAX* gene expression in muscular tissue among the different treated groups compared to the infected non-treated group (Fig. 7).

## Discussion

Trichinosis is a foodborne parasitic disease that can have serious and even fatal effects on individual health if not properly treated [21]. As consumption of FNK seeds at high doses and for long time can induce some toxicological side effects, the dose in the current study was selected based on study by Hassan *et al.* (2023) [7] who stated that FNK seed extract improved the toxic effects of anabolic steroids on cardiac and skeletal muscles and was better than silymarin in relieving the induced histopathological, oxidative stress and biochemical markers alterations. Therefore, different FNK extracts were considered as potentially safer and more effective agents in treating trichinosis.

In the present study, a significant reduction in adult worms and larvae count was observed in all treated groups compared to the infected non-treated controls in both the intestinal and muscular phases. ABZ showed higher activity against adult worms in the intestinal phase, recording a 100% reduction when administered alone or combined with FNK and FNK R. However, in the muscular phase, its activity against encapsulated larvae was enhanced when combined with FNK R (89.4%) and FNK (81.9%) extracts. Similarly, several studies have reported that the activity of ABZ in the muscular phase increased when combined with other therapeutic agents, such as mefloquine, linex, *Zingiber officinale*, platelet-rich plasma, and chitosan nano-lipid loaded with miltefosine [12, 13, 15, 22, 23]. This activity is likely due to the ability of ABZ to inhibit the polymerization of parasite microtubules, depriving the parasite of the energy required for survival [24]. No previous studies have reported the activity of FNK or FNK R extracts against trichinosis.

Concerning histopathological results, similar to previous studies, intestinal sections of infected non-treated mice revealed sections of adult *T. spiralis* and severe pathological changes in the villi, with marked cellular infiltration. Muscular sections of these control mice showed multiple encysted larvae, marked cellular infiltration, and damage to the surrounding muscle fibers [3, 11–13, 15, 23]. These pathological changes were partially improved in mice groups treated separately with ABZ, FNK, and FNK R. However, marked restoration of normal architecture was recorded in the combined ABZ and FNK R-treated group, followed by the combined ABZ and FNK-treated group. The augmented healing effect of ABZ when combined with either FNK or FNK R was similar to previously reported results by El-Wakil *et al.* (2021) [25] and Atta *et al.* (2024) [23], who documented the marked healing effect of ABZ when added to *Annona muricata* (Graviola) leaf extract or linex, respectively.

TGF-β is critical in muscle recovery and repair as it can inhibit muscle regeneration and promote fibrosis. It was observed that levels of TGF-β increased after muscle damage, suggesting that further inhibition of TGF-β could promote muscle regeneration [26]. In the present study, muscular immunohistochemical staining for TGF-β showed marked expression in infected non-treated mice [27], and lower expression was observed after treatment. Furthermore, light expression was noticed in the groups treated with ABZ combined with FNK or FNK R extracts. Similarly, Beshay *et al.* (2020) [28] reported a significant reduction in TGF-β expression in experimental neurotoxocariasis after ABZ treatment. Additionally, Asoka *et al.* (2024) [29] demonstrated that dietary FNK extracts could regulate the TGF-β signaling pathway in induced inflammation in mice with liver cancer.

IFN-γ, released from activated macrophages during trichinosis, plays a pivotal role in the clearance of the parasite [27]. The present results demonstrated elevated serum levels of IFN-γ in infected non-treated controls at both the intestinal and muscular phases, as previously reported [23, 30]. In contrast, the different treatment regimens showed a significant reduction in serum IFN-γ, with a marked decrease after treatment with combined ABZ and FNK or FNK R extracts. Atta *et al.* (2024) [23] and Mahmoud *et al.* (2024) [30] illustrated that ABZ treatment can reduce the levels of IFN-γ in *Trichinella*-infected mice. Furthermore, Yang *et al.* (2022) [31] recorded that FNK supplementation in broilers showed lower expression of IFN-γ levels and other inflammatory cytokines.

Oxidative stress can be induced during the host immune reaction against *Trichinella* infection. It has been found that the antioxidant enzymes reach their maximum levels during the muscular phase of the parasite [32]. The present study observed that the muscular levels of oxidative enzymes, MDA and nitrate, showed a significant increase in infected non-treated mice, while levels of antioxidant enzymes, such as SOD and catalase, showed a significant decrease, as noticed in previous studies [33–35]. Different treatment regimens significantly decreased oxidative enzymes and increased antioxidant enzymes compared to the infected non-treated controls. However, combined ABZ and FNK R demonstrated the best effect in relieving the underlying oxidative stress. Correspondingly, Hamed *et al.* (2022) [33] and Taha *et al.* (2024) [35] observed that the antioxidant effect of ABZ was increased when added to curcumin and silver nanoparticles, respectively. Additionally, a study by Tewari *et al.* (2020) [36] revealed that dietary FNK supplementation in aging mice can regulate the hepatic antioxidant defense enzymes.

The expression of *BAX*, a related mitochondrial apoptotic gene, during trichinosis, is crucial in regulating the differentiation, proliferation, and apoptosis of infected muscle cells during larval encapsulation [37]. The present results showed overexpression of the *BAX* gene in the muscular tissues of infected non-treated mice. In contrast, various treatment modalities resulted in the downregulation of *BAX* expression. El-Samee *et al.* (2024) [38] demonstrated that ABZ treatment against trichinosis resulted in moderate *BAX* gene expression, while the combined use of propolis extract and ABZ showed mild expression. Bafadam *et al.* (2021) [39] illustrated that FNK extract could attenuate *BAX* gene expression in the cardiac tissues of diabetic rats.

## Conclusion

Our findings showed that adding FNK or FNK R to ABZ during the treatment of trichinosis showed a synergistic effect in decreasing parasitic load, modulating the immune response, and restoring normal histological architecture.

## Data Availability

No datasets were generated or analysed during the current study.
